# When Math Hurts: Math Anxiety Predicts Pain Network Activation in Anticipation of Doing Math

**DOI:** 10.1371/journal.pone.0048076

**Published:** 2012-10-31

**Authors:** Ian M. Lyons, Sian L. Beilock

**Affiliations:** 1 Department of Psychology, University of Chicago, Chicago, Illinois, United States of America; 2 Department of Psychology, Western University, London, Ontario, Canada; Université Pierre et Marie Curie, France

## Abstract

Math can be difficult, and for those with high levels of mathematics-anxiety (HMAs), math is associated with tension, apprehension, and fear. But what underlies the feelings of dread effected by math anxiety? Are HMAs’ feelings about math merely psychological epiphenomena, or is their anxiety grounded in simulation of a concrete, visceral sensation – such as pain – about which they have every right to feel anxious? We show that, when anticipating an upcoming math-task, the higher one’s math anxiety, the more one increases activity in regions associated with visceral threat detection, and often the experience of pain itself (bilateral dorso-posterior insula). Interestingly, this relation was not seen during math performance, suggesting that it is not that math itself hurts; rather, the anticipation of math is painful. Our data suggest that pain network activation underlies the intuition that simply anticipating a dreaded event can feel painful. These results may also provide a potential neural mechanism to explain why HMAs tend to avoid math and math-related situations, which in turn can bias HMAs away from taking math classes or even entire math-related career paths.

## Introduction

Math can be difficult. For some, even the mere prospect of doing math is harrowing. Those with high levels of mathematics anxiety (HMAs) report feelings of tension, apprehension, and fear of math [1]. HMAs underperform in math relative to their low-math-anxious counterparts [2] and tend to avoid math and math-related situations, which in turn can bias them away from taking math classes or even entire math-related career paths [3]. But what underlies the actual feelings of dread effected by math anxiety? Are HMAs’ feelings about math merely psychological epiphenomena? Or is their anxiety grounded in simulation of a concrete, visceral sensation – such as pain – about which they have every right to feel anxious? Answering these questions is important for determining how to reverse HMAs’ tendency to avoid math-related situations.

Interoception (one’s sense of the body’s physiological homeostasis [4]) has been shown to increase with heightened levels of anxiety [5] and thus leads to increased sensitivity to physical pain [6], [7]. Here we ask whether simply thinking about (i.e., anticipating) math can elicit a neural pain response in HMAs. Other psychological causes of pain have been reported, such as when one experiences social rejection [8], [9], [10]. Some researchers examining the overlap between social rejection and physical pain have put forth the evolutionary explanation that it is adaptive for a highly social species to place strong deterrents on anti-social behavior [11], [12]. Mathematics, by contrast, is a recent cultural invention, and hence it seems unlikely that a purely evolutionary mechanism would drive a neural pain response elicited by the prospect of doing math. Thus, math anxiety is an ideal test bed for expanding our understanding of how physically innocuous situations might elicit a neural response reflective of actual physical pain.

We hypothesized that subjective ratings of math anxiety would be positively related to activity in regions associated with the experience of pain (e.g., insular cortices [13]) while *anticipating* an upcoming math task. On the surface, one might assume that any pain experiences associated with math anxiety would occur *during* math performance itself: If someone is made anxious by something (in this case, math), then doing that thing may feel painful. However, as mentioned previously, mathematics is a recent cultural invention, so it seems unlikely that pain responses specific to math have been evolutionarily selected for. This means that any observed relation between math anxiety and pain would likely be more dependent upon one’s feelings and worries about math (i.e., their psychological interpretation or anticipation of the event) than something inherent in the math task itself. Given that people have a greater tendency to worry – and have more cognitive resources available to do so – when they are not engaged in a goal-directed task [14], [15], simply anticipating doing math may be most likely to induce a neural pain response among the highly math-anxious.

Our previous results also point to anticipation of math as an important time point to consider. Specifically, we recently demonstrated that variation in HMAs’ neural responses during anticipation of doing math played a large role in explaining how well they actually performed math [16]. Thus, it seems likely that if highly math-anxious individuals show neural responses in regions known to be involved in experiencing pain, it will be in anticipation of an upcoming math task.

Here it is important to point out that the current work and Lyons and Beilock [16] are complementary subsets of the same larger dataset, and that the current results represent novel analyses. The purpose of Lyons and Beilock was to relate activity during the anticipation of doing math to actual, objective math performance. In the current work, we assess how a measure of subjective experience with math (i.e., math anxiety) relates to anticipatory brain activity. Indeed, in Lyons and Beilock, we were careful to control for math anxiety ratings (which is, by contrast, one of the main variables of interest in the current work). We controlled for math anxiety ratings in Lyons and Beilock to show that HMAs who reduced their math-deficits did so as a function of fronto-parietal cue activity even when controlling for within-group (HMA) variation in subjective math anxiety ratings. In the current work, we focus specifically on variation in the subjective experience of math anxiety and its neural correlates for those individuals who profess to have some math anxiety in the first place.

## Methods

All experimental procedures were approved by the University of Chicago Institutional Review Board (protocol 14276A), and all participants gave informed, written consent before participating. Fourteen HMAs and fourteen low math-anxious individuals (LMAs) were identified in a separate prescreening session using the Short Math Anxiety Rating-Scale (SMARS), which measures math anxiety at the trait level. HMAs ranged from above average to very high in math anxiety (range: 38–76, *M* = 49.56) relative to SMARS published norms (*M* = 30.34 [17]). LMAs were below average in math anxiety (range: 5–24, *M* = 15.00). For the SMARS scale, participants are asked to rate how anxious they would be made to feel by 25 math-related situations. Selected examples: ‘Receiving a math textbook’; ‘Walking to math class’; ‘Being given a set of addition problems to solve on paper’; ‘Realizing you have to take a certain number of math classes to meet the requirements for graduation’; ‘Opening a math or statistics book and seeing a page full of problems’.

General trait-anxiety [18] and working-memory (complex reading span [19]) scores were within normal ranges (trait-anxiety: *M* = 32.9, *SD* = 7.9; working-memory: *M* = 45.9, *SD* = 15.8). For general trait anxiety, participants rate how often they agree with 20 situations related to anxiety or calmness. Selected examples: ‘I feel nervous and restless’; ‘I am cool, calm and collected’; ‘I worry too much over something that doesn’t matter’; ‘I make decisions easily’; ‘I feel that difficulties are piling up so that I cannot overcome them’; ‘I get in a state of tension or turmoil as I think over my recent concerns and interests’. For complex working memory span, participants judged the semantic sensibility of a syntactically valid English sentence (e.g., ‘The only furniture Steve had in his first bowl was his waterbed.’) and were then presented with a single letter (which they were instructed to remember). This pattern was repeated 3 to 7 times (i.e., requiring the encoding of a sequence 3 to 7 letters in length), after which subjects were prompted to recall the letter-sequence in the order presented.

Participants completed a word task and math task (block-design) while neural activity was measured using fMRI. Thirty-two blocks of each task-type (16 hard blocks and 16 easy blocks; 4 trials/block) were randomly interleaved and spread over 8 functional runs. In the math task, participants verified whether arithmetic problems of the form (a*b)*−c = d* were correct, where *a*≠*b*, *c*>0, *d*>0. For hard math problems, 5≤*a*≤9, 5≤*b*≤9 (a*b≥30), 15≤*c*≤19; subtracting *c* from a*b always involved a borrow operation; for foil problems, *d*±2. For easy math problems, 1≤*a*≤9, 1≤*b*≤9 (a*b≤9), 1≤*c*≤8; subtracting *c* from a*b never involved a borrow operation; for foil problems, *d*±1.

In the word task, participants verified whether a word, if reversed, spelled an actual word (e.g., reversing the string yrestym generates mytsery, which is not an English word, so participants should respond ‘no’). For the word task, hard trials were seven letters in length; easy trials were four letters in length. Behavioral differences were not found between easy-math and easy-word tasks for either group (Table 1). In contrast, HMA participants performed significantly worse on the hard-math relative to hard-word task, replicating prior research showing that high-math-anxious individuals underperform on difficult math problems relative to difficulty matched non-math tasks [20]. Given that we found behavioral differences only between the hard-word and hard-math tasks, only the hard-blocks are analyzed below.

**Table 1:** Behavioral Data.

HMAs				
	Hard		Easy	
	Math	Word	Math	Word
ER (µ)	24.7	13.1	2.8	2.2
ER (se)	3.3	2.4	0.8	0.5
RT (µ)	3.77	2.90	1.74	1.59
RT (se)	0.16	0.15	0.10	0.09
**LMAs**				
	**Hard**		**Easy**	
	**Math**	**Word**	**Math**	**Word**
ER (µ)	11.1	13.1	3.6	4.0
ER (se)	2.2	2.1	0.9	0.6
RT (µ)	3.03	2.93	1.59	1.51
RT (se)	0.14	0.13	0.05	0.07

Abbreviations: ER: error-rates (percent incorrect); RT: response-times (sec); se: stander-error of the mean.

Crucially, before each task-block, a cue (yellow circle or blue square) indicated whether the math-task or word-task would follow. Fixation-time between cue-offset and block-onset was jittered (2.5–6.5 sec) to separate the respective neural signals generated during the cue-period and task-period. Fixation-time between trial-block-offset and subsequent cue-onset was 18 sec.

MRI data were acquired using a 3 Tesla Philips Achieva scanner with an 8-channel Philips Sense head-coil. A T2^*^-weighted echo-planar imaging sequence was used to acquire functional images covering the whole brain (32 axial slices) with a repetition time (TR) of 2000 ms and an echo time of 25 ms (ascending acquisition; FOV: 240×240×127.5 mm; 80×80×32 matrix; flip angle: 80°). In-plane resolution was 3×3 mm and the slice thickness was 3.5 mm (0.5 mm skip). Signal from the orbital frontal cortex (OFC) and surrounding tissue was recovered using additional volume shimming with a box of 60×60×60 mm centered on the OFC area. This method utilizes multiple ‘pencil beam’ acquisitions to compute shim values (algorithm provided by Philips). High-resolution anatomical images were acquired (axial plane: 300 slices; slice thickness: 1.2 mm, −.6 mm gap; x-y dimensions: 1.04×1.04; FOV: 250×250×180 mm, 240×240×300 matrix) with a standard Philips T1-weighted SENSE-Ref sequence.

All preprocessing steps and whole-brain data analyses were conducted using BrainVoyager QX (version 2.3.1, Brain Innovation, The Netherlands). Functional images were first slice-time-corrected and then motion-corrected using sinc-interpolation. A high-pass GLM (Fourier basis-set) temporal-filter removed fluctuations <2 cycles, which also removed linear temporal drift. Each functional run was then manually aligned to the participant’s 3D anatomical image, both of which were then transformed into Talairach space. Resulting volumetric time-series files were then spatially smoothed with a 6 mm FWHM Gaussian kernel.

Data from all subjects were next submitted to a random-effects GLM [21] with 6 main predictors of interest: math-cue, word-cue, hard and easy math-task-blocks, and hard and easy word-task-blocks. As noted above, we focus on activity during the hard-blocks in the analyses below. In each voxel and for each participant, parameter estimates (βs) for each participant and each condition were generated. Second level analysis was conducted using these voxelwise βs. ANCOVA procedures using these voxelwise betas as inputs were conducted separately for each functional voxel in Matlab. The resulting statistical maps (partial-*r* or *F*-values, where appropriate) were then converted for display in BrainVoyager, wherein they were initially thresholded at p<.005, and subsequently cluster-level corrected for multiple comparisons using a Monte-Carlo simulation procedure [22] with a family-wise false-positive rate α = .01. With respect to region-of-interest (ROI) analyses, for each participant and predictor, ROI-level βs were determined by averaging βs from all voxels comprising the ROI volume in question (for that participant and that predictor). Once extracted, ROI βs were submitted for analysis in SPSS.

In light of recent debate [23], [24] regarding the reporting of correlational values (upon which some of our analyses rest), we believe it is important to emphasize that *r*-values, like any other summary statistic, carry a certain degree of imprecision, which is exacerbated in cases involving relatively few degrees of freedom. For example, with an *r*-value of.7, to say that one has captured 49% of the variance is an incomplete statement. A more correct statement would be to construct 95% confidence intervals around the estimated value of.7 (which depend on one’s degrees of freedom), and then report this range of potentially captured variance. Therefore, in all tables and figures where we report correlation or partial correlation estimates expressed either in terms of standard deviations (*r*-values) or arbitrary units (β-values), we provide standard errors of that estimate as well.

## Results

**Figure 1:**
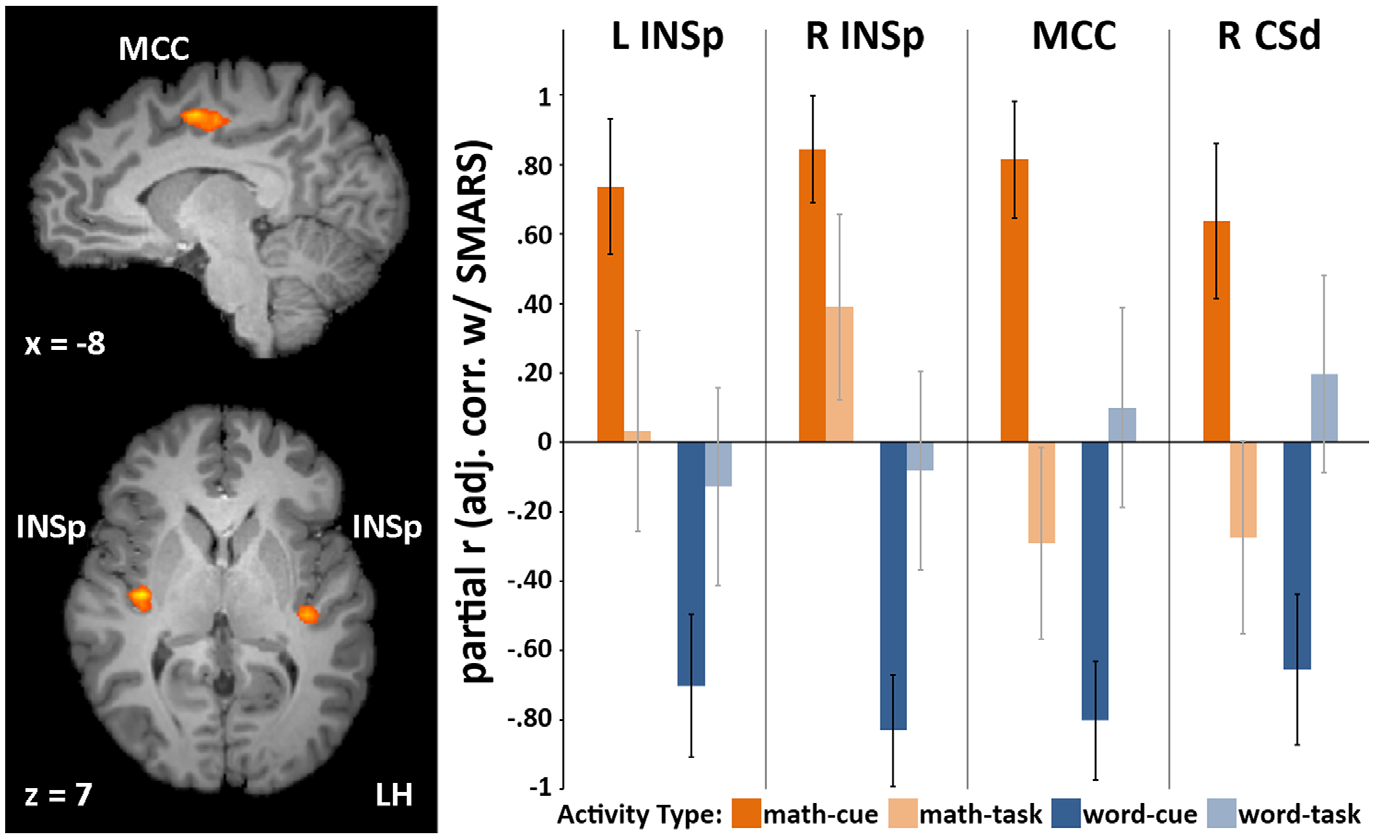
Whole-brain and ROI regression results. **Left:** Regions showing a significant SMARS × 2(Cue: math-cue, word-cue) interaction at the whole brain level (*p*<.005, cluster-corrected at α = .01). INSp: dorso-posterior insula; MCC: mid-cingulate cortex; CSd: dorsal central sulcus (not pictured); see Table 2 (left) for complete region details. **Right:** Multiple-regression adjusted partial *r* correlation coefficients (error-bars represent standard-errors). This is the correlation that remains between the DV (SMARS) and the IV in question, after removing the linear effects of the other three IVs from both variables; IVs = neural-activity: math-cue, math-task, word-cue, word-task. See Table 2 (center) for full regression results. SMARS was chosen as a DV to compare the relative contributions of the various cue and task βs, and in no way implies a causal relation. Note that these bars should not be interpreted as activity levels (i.e., βs relative to baseline), but as partial correlations; see Table 2 for mean βs.

Because we hypothesized that anticipatory activity would be most strongly related to subjective math anxiety ratings in HMAs, we began by submitting HMAs’ cue βs to a SMARS×2 (Cue: math-cue, word-cue) ANCOVA. A whole-brain map of the interaction term was thresholded at *p*<.005 (cluster-level corrected at α = .01). This analysis tested for regions showing a significantly different slope in the relation between SMARS and math-cue-activity and the relation between SMARS and word-cue-activity. Four regions – bilateral dorso-posterior insula (INSp), mid-cingulate cortex (MCC), and a dorsal segment of the right central sulcus (CSd) – showed a significant interaction, driven by a positive relation between SMARS and math-cue-activity and a negative relation between SMARS and word-cue-activity (Figure 1; Tables 2 and 3). Cook’s distances were calculated at the ROI level; none was found to exceed the standard cut-off value of 1. The Cook’s distance of one data-point did exceed .5, but removing it did not change the significance of the results.

**Table 2:** Region Details.

Region	x	y	z	Vol. mm^3^	Predictor	β	se
Left INSp	−39	−23	9	832	math-cue	0.123	0.089
					math-task	−0.39	0.062
					word-cue	0.017	0.074
					word-task	−0.335	0.078
Right INSp	36	−16	9	1584	math-cue	0.076	0.078
					math-task	−0.531	0.061
					word-cue	0.031	0.077
					word-task	−0.565	0.052
MCC	−7	−9	46	1800	math-cue	0.246	0.117
					math-task	−0.081	0.132
					word-cue	0.088	0.123
					word-task	−0.153	0.076
Right CSd	35	−17	43	570	math-cue	0.252	0.101
					math-task	−0.27	0.069
					word-cue	0.19	0.102
					word-task	0.151	0.134

The right-most three columns show mean activity levels in each region.

**Table 3:** Regression Details.

Region	Predictor	*r* partial	*se*	*p*	SMARS × Seg. Int.
Left INSp	math-cue	.737	.195	.010	*F*(1,10) = 8.15
	math-task	.033	.289	.924	*p* = .017
	word-cue	−.702	.206	.016	*F*(1,10) = 4.13
	word-task	−.128	.286	.708	*p* = .069
Right INSp	math-cue	.845	.154	.001	*F*(1,10) = 5.49
	math-task	.389	.266	.216	*p* = .041
	word-cue	−.830	.161	.002	*F*(1,10) = 12.28
	word-task	−.082	.288	.811	*p* = .006
MCC	math-cue	.814	.168	.002	*F*(1,10) = 19.53
	math-task	−.292	.276	.384	*p* = .001
	word-cue	−.802	.172	.003	*F*(1,10) = 17.38
	word-task	.100	.287	.770	*p* = .002
Right CSd	math-cue	.638	.222	.035	*F*(1,10) = 8.93
	math-task	−.275	.278	.413	*p* = .014
	word-cue	−.654	.218	.029	*F*(1,10) = 7.01
	word-task	.197	.283	.561	*p* = .024

Table 3 shows ROI multiple regression results. In each region, SMARS was entered as the dependent measure and math-cue, math-task, word-cue, and word-task activity (βs for each participant) were entered as predictors. SMARS was chosen as a DV to compare the relative contributions of the various cue and task βs, and in no way implies a causal relation. In each region, only math-cue-activity and word-cue-activity remained significant. These results are summarized in the middle section. Note that the *r* partial values should not be interpreted as activity levels (i.e., βs relative to baseline) but as partial correlations; see Table 2 for mean βs. For math-activity, the difference in standardized slopes (*r* partial) between SMARS and cue-activity and SMARS and task-activity was significant in all regions, as assessed by SMARS × 2(Segment: math-cue, math-task) ANCOVAs (the influences of word-cue-activity and word-task-activity were covaried out). *F* and *p* values for these interaction terms are shown in the rightmost column. The same was done for word-activity (grey rows), but with math-cue-activity and math-task-activity covaried out.

We next tested whether the above results were specific to HMAs. In other words, we tested whether HMAs and LMAs fall on the same linear spectrum with respect to the relation between SMARS and cue-activity in regions associated with pain perception. HMA and LMA data were extracted from the four ROIs summarized in Table 2. In each region, we submitted data to a SMARS × 2(Group: HMAs, LMAs; between-subjects variable) × 2(Cue: math-cue, word-cue; within-subjects variable) ANOVA. The main term of interest was the three-way SMARS × Group × Cue interaction, which was significant in all four regions [Left INSp: *F*(1,24) = 10.91, *p* = .003, η^2^ = .313; Right INSp: *F* = 6.00, *p* = .022, η^2^ = .200; MCC: *F* = 18.44, *p*<.001, η^2^ = .434; Right CSd: *F* = 5.45, *p* = .028, η^2^ = .185]. As noted above, there was a strong positive relation between SMARS and math-cue activity in HMAs. For LMAs, however, the relation between SMARS and math-cue-activity (even when controlling only for word-cue-activity, to preserve degrees of freedom and thus better protect against Type II errors) did not obtain significance in any region [expressed as partial *r*s: Left INSp: *r*_p_(11) = −.384, *p* = .195; Right INSp: *r*_p_ = −.119, *p* = .698; MCC: *r*_p_ = −.507, *p* = .077; Right CSd: *r*_p_ = −.379, *p* = .422]. Note that in the cases where the correlation approached significance, it was in fact non-significantly negative. These results are thus consistent with a nonlinear (or perhaps qualitative) distinction between HMAs and LMAs. In particular, the relation between SMARS and math-cue activity was specific to the HMA group. Thus, in the analyses that follow, we have chosen to maintain our theoretical focus on the HMAs.

For HMAs, we next examined the specificity of the relation between SMARS and math-cue-activity in each of these regions by testing whether said relation remained significant when controlling for word-cue-activity, word-performance, math-performance, and trait-anxiety. The relation obtained significance in all regions (*p*s<.05), with the exception of right CSd (*p* = .100). This finding suggests that the relation we observed between math anxiety and math-cue-activity are not accounted for by either generalized anxiety or performance. This latter point is important, because performance did differ between hard-math and hard-word problems (with HMAs, on average, performing worse on the former than the latter, see Table 1). Thus, showing that the relation holds even when accounting for individual differences in performance indicates that the neural responses in the current study aren’t merely an artifact of anticipating having to do a harder task; rather, this response appears to be specific to anticipating doing a math task. Controlling for word-cue activity also indicates that our whole-brain results were not driven solely by word-cue-activity. Interestingly, word-cue-activity remained a significant predictor of SMARS in left INSp and MCC, but in a negative direction. Because the math task and word task were interleaved, HMAs perhaps felt visceral threat or pain when anticipating math, and visceral relief upon recognizing the momentary reprieve of the word task. Admittedly, the result for the word task was unexpected, and this explanation should be viewed as speculative.

For HMAs, we next examined whether the relation between SMARS and brain-activity was specific to the cue-period. When math-*task*-activity and word-*task*-activity were included with math-*cue*-activity and word-*cue*-activity as predictors of SMARS, only math-cue-activity and word-cue-activity remained significant predictors (Figure 1-right; Table 3-center). More conservatively, we tested whether the slope of the relation between SMARS and math-*cue*-activity and that between SMARS and math-*task*-activity were significantly different from each, by testing for a SMARS × 2(Math Segment: cue, task) interaction. This interaction term obtained in all regions (*p*s<.05), even when simultaneously controlling for word-cue-activity and word-task-activity (Table 3-right). Note that the same was true for word activity: the SMARS × Segment interaction obtained when controlling for math cue and task activity (*p*s<.07) (Table 3-right, grey rows). Again, this raises the interesting prospect that, in the context of doing math, anticipating the word task may have served as a kind of refuge, in that, for the moment at least, it meant one didn’t have to do math.

## Discussion

The dorso-posterior insula (INSp) and mid-cingulate cortex (MCC) are implicated in pain perception. Nocioceptive-specific lamina I projections synapse in posterior-ventromedial thalamus (VMpo [25], [26]), and outputs from VMpo terminate in mid-posterior dorsal INS [4]. Direct stimulation of INSp in humans yields pain responses [27]. Neuroimaging evidence in humans supports somatotopically organized contralateral responses to pain-stimulation in INSp [10], [28], [29], [30]. In a recent case study, seizures likely emanating from a dysplasia in right INSp propagated to other pain-related areas (including MCC) and were associated with strong left-lateralized pain sensation; direct stimulation of only INSp generated pain responses akin to those experienced during spontaneous seizure attacks [31]. Mid-posterior INS functionally [32] and anatomically [33] connects with dorsal MCC. Interestingly, MCC in our study showed stronger connectivity with INSp (bilaterally) for cue-activity relative to task-activity (left: *z* = 3.05, *p* = .002; right: *z* = 2.95, *p* = .003). In sum, high levels of math anxiety predict increased pain-related activity during anticipation of doing math, but not during math performance itself.

Although we feel that a pain-related experience is the best functional interpretation of INSp activity, it is important to point out that our interpretation is, in essence, a form of reverse-inference [34], and that the INSp activity we found could be reflective of something else. For example, it has been suggested that INSp activity is not so much reflective of nocioception, but rather reflects detection of events that are salient for (e.g., threatening to) bodily integrity, regardless of the input sensory modality ([35]; though see [31] and [36] for evidence to the contrary; see also [37] and [38] for recent meta-analyses on insular cortical function). We believe the majority of the evidence in the literature supports our interpretation that INSp activation reflects pain perception (per our discussion in the previous paragraph and additional evidence discussed in the next paragraph). That said, even if one were to adopt to view that INSp activity reflects the detection of a salient (potentially threatening) bodily event, this nonetheless carries important implications for understanding the subjective experience of math anxiety. If the experience of math anxiety is grounded in a visceral, aversive bodily reaction (whether there is an accompanying pain percept or not), this visceral response poses a clear mechanism that may explain the observation that HMAs tend to avoid math and math-related situations [3].

In addressing the issue of reverse-inference more broadly, one potential method some researchers have adopted is to use a functional localizer. We did not adopt this approach here because it does not circumvent the potentially faulty logic of reverse inference: If a given region supports multiple functions, it will still coactivate in a single sample or subject. A more recent (and we believe superior) proposal is to treat the problem in a Bayesian framework [39] – to compute, using a continuously updated meta-analytical approach, how a given human semantic term (e.g., ‘memory’, ‘emotion’, etc.) allows one to selectively predict (i.e., calculate the posterior probability of) activity in a given brain area. This concept has been implemented at neurosynth.org (for methodological details and verification procedures, see [40]). Thus, in our case, one can calculate the probability with which studies that use the word ‘pain’ show activity in particular brain areas relative to studies that do not use that term. In other words, within the extant literature, to what degree does activity in posterior insula selectively predict the occurrence of the term ‘pain’? We used this method to calculate the selective posterior probability for the term ‘pain’ at each of the coordinates corresponding to the four regions in Table 2. MCC and right CSd were not selective for pain (*z*s = 0). However, both INSp regions were highly selective for pain (right: *z* = 8.05, left: *z* = 4.51; both *p*s<5E−5, two-tailed). Thus, a data-driven, meta-analytical approach suggests a high degree of selectivity for pain-related activity in bilateral posterior insular cortices. This supports our functional interpretation of the INSp regions shown in the current study: greater subjective math anxiety ratings in HMAs are related to greater activity in regions associated with the experience of visceral pain (during anticipation of an impending math task).

In sum, we provide the first neural evidence indicating the nature of the subjective experience of math anxiety. In particular, higher subjective ratings of math anxiety predicted greater activation in INSp when anticipating a math task. Here it is important to note that previous research on the overlap between pain processing and psychological experience of social rejection has focused primarily on the actual experience of being rejected. Our data go beyond these results and suggest that even *anticipating* an unpleasant event is associated with activation of neural regions involved in pain processing. Further, leading explanations for the overlap between social rejection and physical pain have tended to rely on evolutionary mechanisms [11], [12], [41]. Because it seems unlikely that a purely evolutionary mechanism would drive a neural pain response elicited by the prospect of doing math (as math is a recent cultural invention), this opens up the prospect that pain network activation is not limited to situations directly related to evolved pain responses.

Interestingly, the relatively posterior regions we find here in the insular cortices (INSp) are anatomically quite close to those activated during severe social rejection experiences (e.g., viewing images that allude to adverse relationship break-ups [10]), rather than the more anterior prefrontal regions that are activated in less severe rejection situations (e.g., being socially rejected by someone you don’t know [8]). INSp is thought to underlie direct sensory experience of pain, whereas more anterior insula cortex areas to be implicated in the affective component and regulation of pain responses [11], [42]. Kross et al. [10] also demonstrated that INSdp voxels active when experiencing severe social rejection overlapped with INSdp voxels active during the sensory experience of physical pain. Our work extends Kross et al.’s findings by showing that, when highly math-anxious individuals *merely anticipate doing a learned, culturally acquired activity* (math), regions involved in the sensation of pain are active as well.

When anticipating an upcoming math-task, the higher one’s math anxiety, the more one increases activity in regions associated with bodily threat detection and the experience of visceral pain itself (INSp). Given our findings were specific to cue-activity, it is not that math itself hurts; rather, merely the anticipation of math is painful. Anticipatory anxiety about math is grounded in the simulation of visceral threat and even pain. These results also provide a potential neural mechanism to explain the observation that HMAs tend to avoid math and math-related situations, which in turn can bias HMAs away from taking math classes or even entire math-related career paths [3].
